# Supplementary material for: Heterogeneous preferences and asymmetric insights for AI use among welfare claimants and non-claimants
Source: Nat Commun. 2025 Jul 29;16:6973. doi: 10.1038/s41467-025-62440-3 (PMC12307907; doi:10.1038/s41467-025-62440-3)

Supplementary Information for

Heterogeneous preferences and asymmetric insights for AI use among welfare claimants and non-claimants

Mengchen Dong[1]*, Jean-François Bonnefon[2], and Iyad Rahwan[1]

[1] Center for Humans and Machines, Max Planck Institute for Human Development, Berlin, Germany

[2] Department of Social and Behavioral Sciences, Toulouse School of Economics, Centre National de la Recherche Scientifique (Toulouse School of Management Research), University of Toulouse Capitole, Toulouse, France

This PDF file includes:

Supplementary Methods
Supplementary Notes
Supplementary Fig. 1 to 8
Supplementary Tables 1 to 4

Supplementary Fig. 1.

Sample stimuli of the US representative-sample study ($N = 987$) across different conditions.

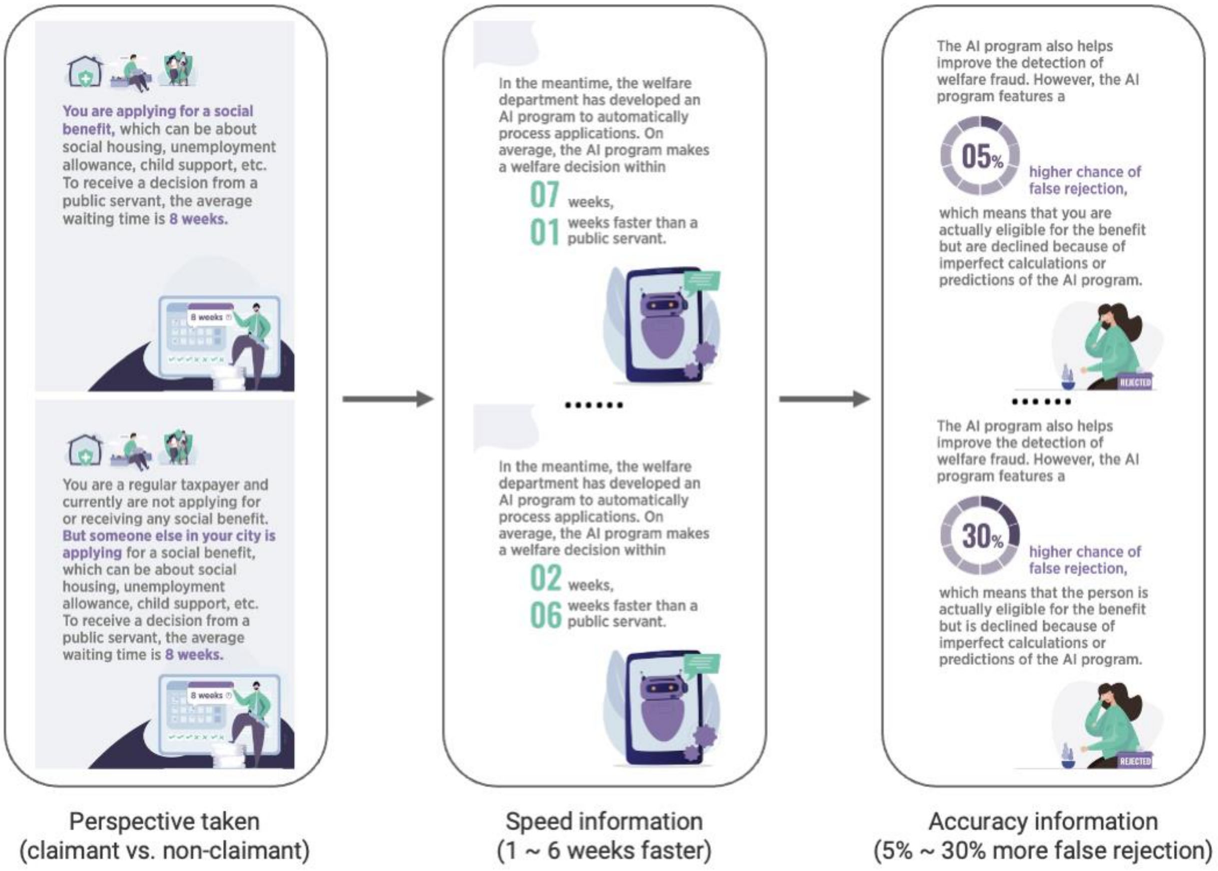

Supplementary Fig. 2.

Sample stimuli of the balanced-sample UK study (*N* = 1,462) across different conditions.

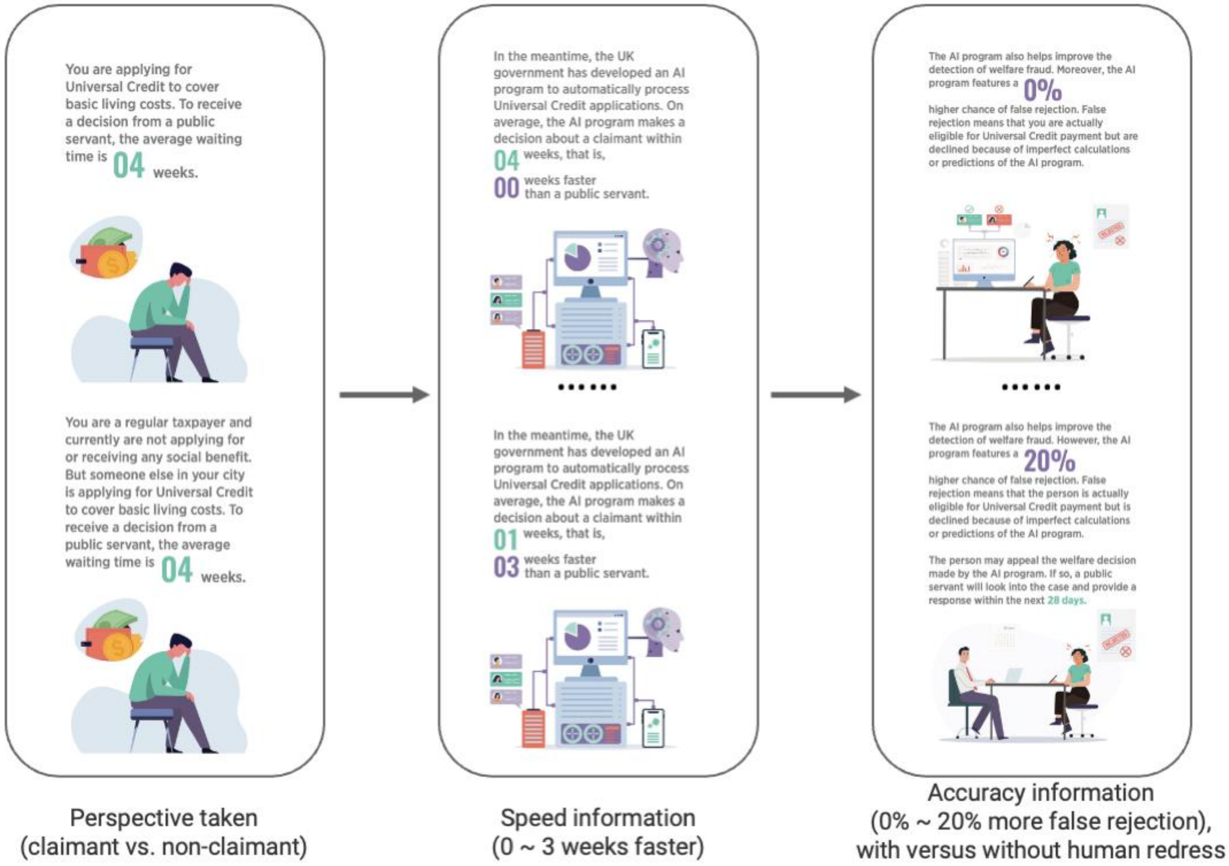

Supplementary Fig. 3.

Sample stimuli of the balanced-sample US conjoint study ($N = 800$) across different conditions.

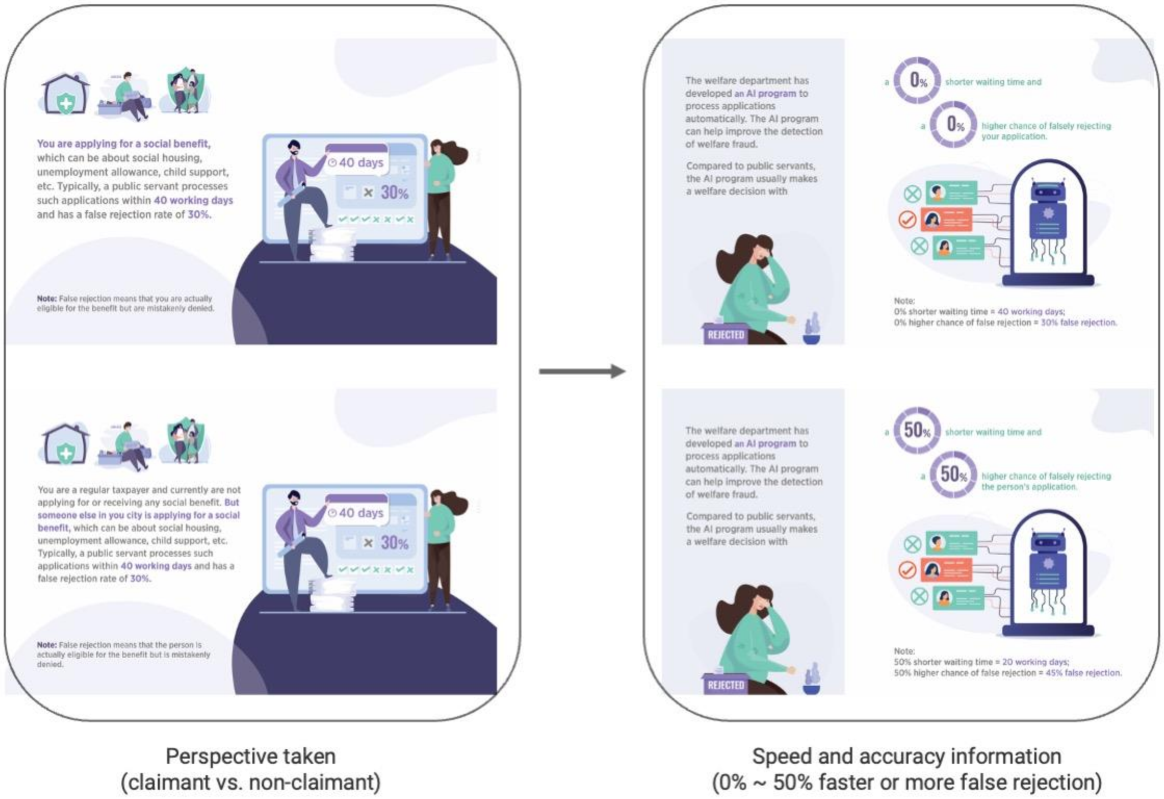

A. Supplementary Methods
A.1 The US representative-sample study
A.1.1 Informed consent

Study Information and Statement of Informed Consent for Adult Participants

Thank you for your interest in participating in our *Artificial Intelligence in Social Welfare* study. Please take your time to read this text carefully. If you have any questions, we are happy to answer them. By signing this form, you agree that you have read the information presented and that you are willing to participate in the study.

1. Aim of the study
In the *Artificial Intelligence in Welfare* study, we want to investigate how people feel about Artificial Intelligence systems making social welfare decisions when they operate at different speeds (efficiency) and with different error rates (accuracy).

2. Procedure and content of the study
To do this, we will ask you to think of yourself as either a taxpayer or a welfare recipient. After a short warm-up session to familiarize yourself with the task format, you will be presented with 36 short descriptions, with different information about a welfare AI system's efficiency and accuracy. After reading each description, you will be asked one question about your preference for the AI or a public servant to handle the welfare application.
We will also ask you to provide us with some background information about your age, sex, ethnicity, political ideology, income, and education level.

3. Are there any risks involved?
There are no risks involved in this research.

4. What will happen to the information and data collected?
This study is a research project of the Max Planck Institute (MPI) for Human Development. The data collected will be used for research purposes only.
No personal contact data (name, address, phone number, email address, etc.) will be recorded. We use your IP address to determine the country from which you access the site, but we do not store the full IP address itself. If you choose to participate in the study, we will link broad information about your age, sex, political ideology, income and education level to your answers throughout the survey. The anonymized study data may be made publicly accessible via research databases or scientific publications. The publicly accessible data may also be used for purposes going beyond this particular study.
The study data (but not personal contact data) may be shared with cooperation partners for collaborative analysis. The study data may also be made publicly accessible via research data bases or scientific publications (typically via the Internet). This makes it possible for other researchers to check or replicate the results of the study and enhances the quality of scientific research. The study data may also be used for new research questions going beyond the purposes of this particular study. Please note that once study data are publicly accessible, its further distribution by others cannot be ruled out, and that this is beyond the area of influence or responsibility of the MPI for Human Development. Therefore, as a matter of principle, study

data are only transferred or made publicly accessible without personal contact data. Data in which persons are identifiable can be made publicly accessible only if the participant agrees to this use by signing a separate consent form.

5. Participation is voluntary

Participation in this study is voluntary. You may withdraw from the study at any time without giving any reason and without any negative consequences. You may also withdraw your consent to data processing and usage at any time with effect for the future and without negative consequences. To do this, please contact dong@mpib-berlin.mpg.de.
Please note that since no personally identifiable information is collected, it will not be possible to link your study data to your person. Hence it will not be possible to withdraw your consent to further processing your study data as described above once the study is completed.

6. Consent

This document has informed you about participation in the *Artificial Intelligence in Welfare* study. The study involves a single session and some background information questions that takes about 12 mins to complete. You will receive compensation of £1.6 for your participation in the study. If you decide to terminate your participation before the end of the study, you will receive compensation of £0.

○ By checking this box, you are indicating that you have read and understood the conditions outlined above and CONSENT to participate in the study, and agree to the collection, storage, and use of your data as described above. (1)

○ By checking this box, you are indicating that you have read and understood the conditions outlined above and DO NOT CONSENT to participate in the study, and agree to the collection, storage, and use of your data as described above. (2)

A.1.2 Demographic Information and welfare status

Before starting, please provide some basic information about yourself:
Please fill your Prolific ID in the blank below:

____

What is your age?

____

What is your sex?

- ◯ Male
- ◯ Female

What ethnic group do you belong to?

- ◯ White
- ◯ Black
- ◯ Asian
- ◯ Mixed
- ◯ Other

Here is a 7-point scale on which the political views that people might hold are arranged from extremely liberal (left) to extremely conservative (right).

Where would you place yourself on this scale?

	Extremely liberal (left)	Extremely conservative (right)
	0 1 2 3	4 5 6 7
Political Ideology ()		

Which of these is the highest level of education you have completed?

- ○ No formal qualifications
- ○ Secondary education (e.g. GED/GCSE)
- ○ High school diploma/A-levels
- ○ Technical/community college
- ○ Undergraduate degree (BA/BSc/other)
- ○ Graduate degree (MA/MSc/MPhil/other)
- ○ Doctorate degree (PhD/other)

What is your total household income per year, including all earners in your household (after tax)?

- ○ Less than $10000
- ○ $10000–$15999
- ○ $16000–$19999
- ○ $20000–$29999
- ○ $30000–$39999
- ○ $40000–$49999
- ○ $50000–$59999
- ○ $60000–$69999
- ○ $70000–$79999
- ○ $80000–$89999
- ○ $90000–$99999
- ○ $100000–$149999

◯ More than $150000

Are you currently applying for or receiving any kind of social welfare benefits (e.g., disability, unemployment, childcare, retirement, etc.)?

◯ Yes

◯ No

A.1.3 Study Introduction and perspective manipulation

This study aims to investigate how people feel about Artificial Intelligence (AI) systems making social welfare decisions.

Artificial intelligence (AI) refers to systems that display intelligent behaviour by analysing their environment and taking actions – with some degree of autonomy – to achieve specific goals (The European Commision, 2018).

You will be asked to put yourself in a particular social role, and then evaluate 36 different descriptions of welfare AI systems that operate at different speeds (efficiency) and with different error rates (accuracy).

Please put yourself in the shoes of a **WELFARE RECIPIENT**.

As a welfare recipient, part of your living depends on social welfare benefits provided by the government. These benefits are funded by other taxpayers who pay a standard rate of their income to the government on a regular basis.

After reading the above information, please answer:

You should put yourself in the shoes of ......

◯ A taxpayer

◯ A public servant

◯ A welfare recipient [proceed only after providing this answer]

Please put yourself in the shoes of a **TAXPAYER**.

As a taxpayer, you pay a standard rate of your income to the government on a regular basis. The government uses a portion of your and other taxpayers' money to provide social welfare benefits to other people in need.

After reading the above information, please answer:

You should put yourself in the shoes of ......

- ◯ A taxpayer [proceed only after providing this answer]
- ◯ A public servant
- ◯ A welfare recipient

A.1.4 Two exercise trials, random order (taxpayer perspective, for example)

Before starting the main task, you will first familiarize yourself with the task format.

On the next few pages, you will read two sample descriptions of welfare AI programs and answer some questions. You will have a chance to review your answers.

[three cards: perspective + 0 week faster + 50% more false rejections]

[three cards: perspective + 7 weeks faster + 1% more false rejections]

[Both followed by four comprehension check questions and one preference question. Participants can only proceed after providing correct answer to the check questions.]

How long does it take to receive a welfare decision from a public servant?

- ◯ 1 week
- ◯ 2 weeks
- ◯ 3 weeks
- ◯ 4 weeks
- ◯ 5 weeks
- ◯ 6 weeks
- ◯ 7 weeks
- ◯ <u>8 weeks [proceed only after providing this answer]</u>

How many weeks FASTER is the AI program than a public servant?

- ◯ 0 week
- ◯ 1 week
- ◯ 2 weeks
- ◯ 3 weeks
- ◯ 4 weeks
- ◯ 5 weeks
- ◯ 6 weeks
- ◯ 7 weeks
- ◯ 8 weeks

How much HIGHER is the error rate of the AI program than a public servant?

- ◯ 1%

- ◯ 10%
- ◯ 20%
- ◯ 25%
- ◯ 30%
- ◯ 50%

Which below statement about false rejection is correct?

- ◯ False rejection means that the person is eligible for the benefit and is accepted.
- ◯ False rejection means that the person is eligible for the benefit but is rejected. [proceed only after providing this answer]
- ◯ False rejection means that the person is ineligible for the benefit but is accepted.
- ◯ False rejection means that the person is ineligible for the benefit and is rejected.

To what extent do you prefer a public servant or the AI program to handle THE PERSON'S welfare application?

	Definitely a public servant — No preference — Definitely the AI program<br>0 10 20 30 40 50 60 70 80 90 100
As a taxpayer, you prefer: ()	[slider]

Below you can see your indicated preferences to the two different states of the welfare AI program.

Please review your answers and make any changes as you wish.

[The three cards appear again together with the preference question, anchoring the answer at participants' previous response']

Are you ready to proceed, after familiarizing yourself with the two examples above?

○ Yes. I am ready to move forward to the main task.

A.1.5 The 36 official trials, random order (taxpayer perspective, for example)

You will start the main task from the next page.

You will read another 36 descriptions of welfare AI programs, with different information about their efficiency and accuracy.

You will NOT be given a chance to change your answers anymore. So please read them carefully, evaluate them independently, and answer ONE question about your preference following each description.

[Three cards followed by preference questions]

To what extent do you prefer a public servant or the AI program to handle THE PERSON'S welfare application?

	Definitely a public servant	No preference	Definitely the AI program
	0 10 20 30	40 50 60	70 80 90 100
As a taxpayer, you prefer: ()			

A.2 The UK balanced-sample study
A.2.1 Informed Consent

[Similar to the US study, with minor differences in Sections 2 and 6.]

2. Procedure and content of the study
To do this, we will ask you to think of yourself as either a taxpayer or a welfare claimant. After a short warm-up session to familiarize yourself with the task format, you will be presented with 20 short descriptions, with different information about how a welfare AI system operates and its speed and error rate. After reading each description, you will be asked two questions about (1) your preference for the AI or a public servant and (2) your trust in your government if the AI is used, in handling welfare applications.
We will also ask you to provide us with some background information about your age, sex, ethnicity, political ideology, education level, and welfare status.

6. Consent
This document has informed you about participation in the *Artificial Intelligence in Social Welfare* study. The study involves some background information questions and a single session that takes about 12 mins to complete. You will receive compensation of £1.6 for your participation in the study. You will also have a chance to win a bonus (£0 ~ £1.2) depending on your role and performance. If you decide to terminate your participation before the end of the study, you will receive compensation of £0.

A.2.2 Demographic Information and welfare questions

[Similar to the US study; different questions are listed below.]

Are you a recipient of Universal Credit?
(For more information about Universal Credit, see https://www.gov.uk/universal-credit)

○ Yes

○ No

Display This Question:

If Are you a recipient of Universal Credit? = No

Do you have close families or friends who are currently applying for or receiving social benefits from Universal Credit?

○ Yes

○ No

Display This Question:

If Are you a recipient of Universal Credit? = No

Have you ever applied for or received any other social benefits to meet basic live expenses?

○ Yes

○ No

Display This Question:

If Are you a recipient of Universal Credit? = No

To what extent do you think you are unlikely or likely to apply for Universal Credit in the near future?

	Very unlikely	Neutral	Very likely
	0 10 20 30	40 50 60	70 80 90 100
Likelihood of applying ()			

A.2.3 Study Introduction and perspective manipulation

This study aims to investigate how people feel about Artificial Intelligence (AI) systems making social welfare decisions.

Artificial intelligence (AI) refers to systems that display intelligent behaviour by analysing their environment and taking actions – with some degree of autonomy – to achieve specific

goals (The European Commision, 2018).

You will be asked to put yourself in a particular social role, and then evaluate 20 different descriptions of welfare AI systems that operate at different speeds (efficiency) and with different error rates (accuracy).

Please put yourself in the shoes of <u>a CLAIMANT of Universal Credit</u>. Universal Credit is a welfare payment provided by the UK government (for more information about Universal Credit, see https://www.gov.uk/universal-credit**).**

As a claimant of Universal Credit, you are usually unemployed (or earning a low income). You rely on the benefit to meet basic living expenses. The allowance is provided by the UK government and potentially funded by other taxpayers who pay a standard rate of their income to the government on a regular basis.

After reading the above information, please answer:

You should put yourself in the shoes of ......

- ◯ A taxpayer
- ◯ A public servant
- ◯ <u>A claimant of Universal Credit [proceed only after providing this answer]</u>

Please put yourself in the shoes of <u>a TAXPAYER in the UK</u>.

As a taxpayer in the UK, you pay a standard rate of your income to the UK government on a regular basis. The government uses a portion of your and other taxpayers' money to support other citizens in need. For example, Universal Credit provides a welfare payment to people who are either unemployed or earning a low income (for more information about Universal Credit, see https://www.gov.uk/universal-credit**).**

After reading the above information, please answer:

You should put yourself in the shoes of ......

- ○ A taxpayer [proceed only after providing this answer]
- ○ A public servant
- ○ A claimant of Universal Credit

A.2.4 Two exercise trials, random order (claimant condition, for example)

[Similar to Study 1, with different card information including human redress and an additional trust question after each trial.]

[three cards: perspective + 0 week faster + 40% more false rejections]

[three cards: perspective + 3 weeks faster + 1% more false rejections]

How long does it take to receive a welfare decision from a public servant?

- ○ 1 week
- ○ 2 weeks
- ○ 3 weeks
- ○ 4 weeks [proceed only after providing this answer]

How many weeks FASTER is the AI program than a public servant?

- ○ 0 week
- ○ 1 week
- ○ 2 weeks
- ○ 3 weeks

How much HIGHER is the false rejection rate of the AI program than a public servant?

- ○ 0%
- ○ 5%
- ○ 10%
- ○ 15%
- ○ 20%
- ○ 40%

Which below statement about false rejection is correct?

- ○ False rejection means that you are eligible for the benefit and are accepted.
- ○ False rejection means that you are eligible for the benefit but are rejected. [proceed only after providing this answer]
- ○ False rejection means that you are ineligible for the benefit but are accepted.
- ○ False rejection means that you are ineligible for the benefit and are rejected.

To what extent do you prefer a public servant or the AI program to handle YOUR welfare application?

Definitely a public servant	No preference	Definitely the AI program

0 10 20 30 40 50 60 70 80 90 100

As a welfare claimant, you prefer: ()	

If the UK government decided to replace some public servants with the AI program in handling welfare applications, would your trust in the government decrease or increase?

Decrease a lot	Neither decrease nor increase	Increase a lot

0 10 20 30 40 50 60 70 80 90 100

As a welfare claimant, your trust would: ()	

A.2.5 The 20 official trials, random order (claimant perspective, for example)

You will start the main task from the next page. You will read another 20 descriptions of welfare AI programs, with different information about their speed and error rate, and answer TWO questions following each description. You will NOT be given a chance to change your answers anymore.

Please remember to put yourself in the shoes of <u>a CLAIMANT of Universal Credit</u>, read the descriptions carefully, and evaluate each of them independently.

Your answer to each question will be compared with that of the group who are currently applying for or receiving Universal Credit. Each answer will be considered correct when it falls within ±5 points (on the 100-point scale) of their group average and redeem £0.03 bonus pay. Put differently, you will have a chance to win £0 to £1.2 bonus given your answers to 40 questions (i.e., 20 descriptions, each with two questions). You will receive the bonus pay within two weeks upon completion of the study.

[Three cards followed by preference and trust questions]

To what extent do you prefer a public servant or the AI program to handle YOUR welfare application?

Definitely a public servant	No preference	Definitely the AI program

0 10 20 30 40 50 60 70 80 90 100

As a welfare claimant, you prefer: ()	

If the UK government decided to replace some public servants with the AI program in handling welfare applications, would your trust in the government decrease or increase?

	Decrease a lot	Neither decrease nor increase	Increase a lot
	0 10 20 30	40 50 60	70 80 90 100
As a welfare claimant, your trust would: ()			

A.2.6 Manipulation check of human redress

To what extent do you believe that you can appeal to public servants if you are not satisfied with the welfare decision made by the AI program?

	Not at all	Moderately	Very much
	0 10 20 30	40 50 60	70 80 90 100
Belief about the chance to appeal ()			

A.3 The US balanced-sample conjoint study
A.3.1 Informed Consent

[Similar to the first US study]

A.3.2 Welfare questions to preselect claimants and non-claimants

***Non-claimants are selected to meet all three standards: (1) answering "No" to the first question, (2) answering "I am NOT applying for or receiving any welfare benefits" to the second question, and (3) answering "Not at all" to the third question.**
***Claimants are selected to meet all three standards: (1) answering "Yes" to the first question, (2) answering any choice other than "I am NOT applying for or receiving any welfare benefits" to the second question, and (3) answering any choice other than "Not at all" to the third question.**

Are you currently applying for or receiving any kind of social welfare benefits (e.g., disability, unemployment, childcare, retirement)?

○ Yes

○ No

Which social welfare benefits are you currently applying for or receiving?

- Supplemental Security Income (SSI)
- Supplemental Nutrition Assistance Program (SNAP)
- Special Supplemental Nutrition Program for Women, Infants, and Children (WIC)
- Temporary Assistance for Needy Families (TANF), including Pass through Child Support
- General Assistance (GA)
- Social security (self and on behalf of a dependent child)
- Department of Veterans' Affairs benefits (except Veteran's pension)
- Unemployment insurance compensation
- I am applying for or receiving a welfare benefit that is not listed above
- I am NOT applying for or receiving any welfare benefits

In your life time, how long have you been receiving any kind of social welfare benefits?

- Not at all
- One month to one year
- One to two years
- Two to three years
- Three to five years
- Five to ten years
- Longer than 10 years

A.3.3 Perspective manipulation

[Similar to the first US study]

A.3.4 Two exercise trials, random order (taxpayer perspective, for example)

Now, putting yourself in the shoes of a taxpayer, you need to make a choice between the two AI programs:

[Waiting time: **0%** shorter False rejection rate: **50%** higher VS Waiting time: **50%** shorter False rejection rate: **0%** higher]

[Waiting time: **0%** shorter False rejection rate: **0%** higher VS Waiting time: **50%** shorter False rejection rate: 5**0%** higher]

[Both preceded by four comprehension check questions. Participants can only proceed after providing correct answer to the check questions.]

Which AI program would you prefer?

A.3.5 The 30 official trials, random order (claimant perspective, for example)

--- You will start the main task from the next page. ---

Before that, please remember the basic information below about how long and how accurate public servants make welfare decisions. That is, a public servant typically processes welfare applications within **40 working days** and has a **false rejection rate of 30%**.

In the main task, you will read 30 pairs of welfare AI programs and their performance as compared to public servants. Your task is to make a choice for each pair, putting yourself in the shoes of a WELFARE CLAIMANT.

You will NOT be given a chance to revise your answers anymore. So please read and choose carefully. [See Supplementary Fig. 4 below the interface for a sample trial]

Supplementary Fig. 4.

Choice interface of a sample trial in the balanced-sample US conjoint study (N = 800).

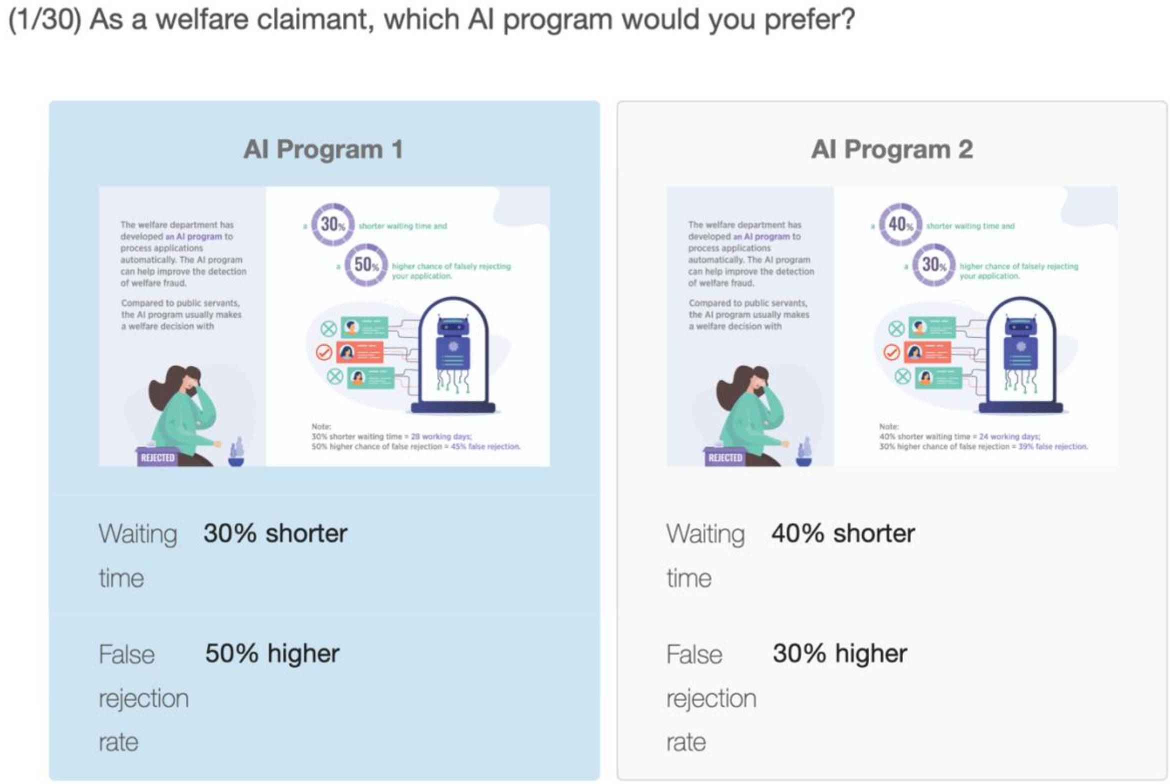

B. Supplementary Notes

B.1 The US representative-sample study

Overall, we had 987 participants, including 473 males and 514 females based on self-report. Using M-plus, we conducted the latent profile analysis on participants who indicated preference from their inherent perspective ($N = 506$; i.e., claimants taking claimant perspective, and non-claimants taking taxpayer perspective;). We explored the profile number ranging from 1 to 5, and reported their model fit metrics in **Supplementary Table 1**. As also shown in the elbow plot in **Supplementary Fig. 5**, there was no strong support for a particular number of profiles, for example, a three- or five-profile model. And the model performance seems to continue improving by extracting larger numbers of profiles with less theoretically meaningful interpretations.

Despite so, for respectively three-, four-, and five-profile models, we present the descriptive information of demographic features related to vulnerable characteristics (e.g., age, income, welfare status) in **Supplementary Table 2**, and the preference data clustered by the latent profiles in **Supplementary Fig. 6**.

B.2 The UK balanced-sample study

Manipulation check. The human redress manipulation was successful. Participants had a stronger belief about the chance to appeal in the human redress ($n = 732$; $M = 78.1$, $SD = 24.6$) than in the no redress condition ($n = 730$; $M = 63.2$, $SD = 27.5$; $\beta = 0.20$, $p < .001$).

Overall, we had 1462 participants, including 624 males, 817 females, and 21 self-identified as other, based on self-report. As in the US study, we also conducted the latent profile analysis on participants who indicated preference from their inherent perspective ($N = 739$; i.e., claimants taking claimant perspective, and non-claimants taking taxpayer perspective). We explored the profile number ranging from 1 to 5, and reported their model fit metrics in **Supplementary Table 3**. As also shown in the elbow plot in **Supplementary Fig. 7**, there was no strong support for a particular number of profiles, for example, a three- or five-profile model. And the model performance seems to continue improving by extracting larger numbers of profiles with less theoretically meaningful interpretations.

Still, for respectively three-, four-, and five-profile models, we present the descriptive information of demographic features related to vulnerable characteristics (e.g., age, income, welfare status) in **Supplementary Table 4**, and the preference data clustered by the latent profiles in **Supplementary Fig. 8**.

B.3 The US balanced-sample conjoint study

We had 800 participants. Among these participants, 424 self-reported as males, 370 as females, 4 as other, and 2 preferred not to say.

Supplementary Table 1.

Model parameters of the latent profile analysis in the US study (*N* = 506).

Model with *N* of profile(s)	1 profile	2 profiles	3 profiles	4 profiles	5 profiles
LogLik	-86182,5	-81172,1	-78987,8	-78007,9	-77570
AIC	172508,9	162562,2	158267,6	156381,8	155579,9
AWE	173475,5	164026,6	160229,8	158841,7	158537,7
BIC	172813,2	163022,9	158884,7	157155,2	156509,8
CAIC	172885,2	163131,9	159030,7	157338,2	156729,8
CLC	172366,9	162346,2	157977,6	156017,7	155141,9
KIC	172583,9	162674,2	158416,6	156567,8	155802,9
SABIC	172584,7	162676,9	158421,3	156574,4	155811,5
ICL	-172813	-163025	-158893	-157174	-156537
Entropy	1	0,988423	0,98309	0,973704	0,968276
prob_min	1	0,996995	0,990551	0,977831	0,969142
prob_max	1	0,998163	0,995187	0,990765	0,989715
n_min	1	0,464427	0,262846	0,201581	0,150198
n_max	1	0,535573	0,448617	0,328063	0,274704
BLRT_val	N/A	10020,72	4368,588	1959,814	875,8508
BLRT_p	N/A	0,009901	0,009901	0,009901	0,009901

Supplementary Table 2.

Demographic descriptive information of 3 to 5 latent groups for the US representative-sample study ($N = 506$).

		Age	Sex (as females)	Income Mean (SD)	Welfare status (as claimants)
Three-profile model	Average	44.3 (16.0)	51.4%	7.7 (3.7)	20.9%
	Group 1 (26%)	41.0 (15.7)	47.4%	8.0 (3.8)	18.0%
	Group 2 (45%)	42.8 (15.7)	54.6%	7.9 (3.7)	18.1%
	Group 3 (29%)	49.7 (15.7)	50.0%	7.0 (3.5)	28.1%
Four-profile model	Average	44.3 (16.0)	51.4%	7.7 (3.7)	20.9%
	Group 1 (20%)	40.2 (15.8)	45.1%	8.0 (3.8)	17.6%
	Group 2 (33%)	42.2 (15.5)	53.0%	8.0 (3.7)	17.5%
	Group 3 (25%)	44.8 (15.5)	58.4%	7.9 (3.7)	17.6%
	Group 4 (22%)	50.5 (15.9)	46.9%	6.7 (3.4)	32.7%
Five-profile model	Average	44.3 (16.0)	51.4%	7.7 (3.7)	20.9%
	Group 1 (18%)	41.1 (15.9)	42.7%	8.0 (3.8)	20.2%
	Group 2 (27%)	41.6 (15.8)	50.4%	8.0 (3.7)	16.5%
	Group 3 (24%)	43.9 (15.6)	60.3%	8.0 (3.7)	19.0%
	Group 4 (15%)	45.5 (15.4)	56.6%	7.1 (3.5)	18.4%
	Group 5 (16%)	52.0 (15.7)	44.4%	6.9 (3.6)	34.6%

Supplementary Fig. 5.

Elbow plot for model selection in the US representative-sample study (N = 506).

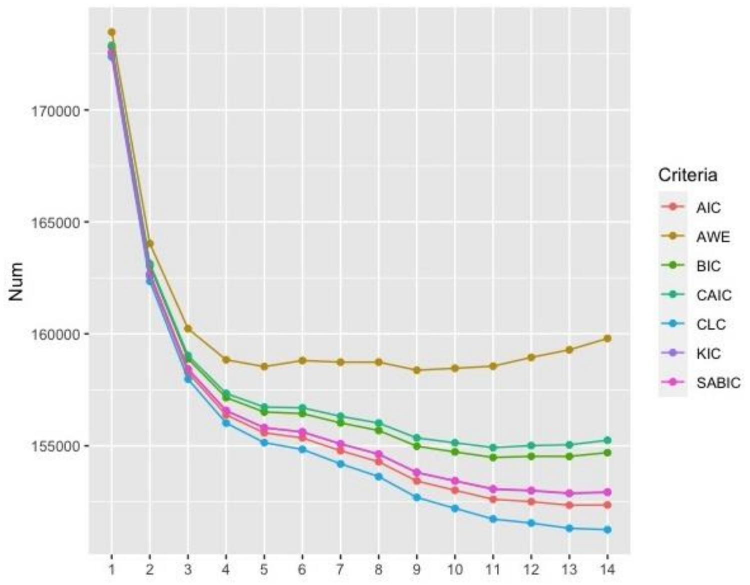

Supplementary Fig. 6.

Preference data clustered by latent profiles in the US representative-sample study (N = 506; the number of latent profiles ranged from 3 to 5).

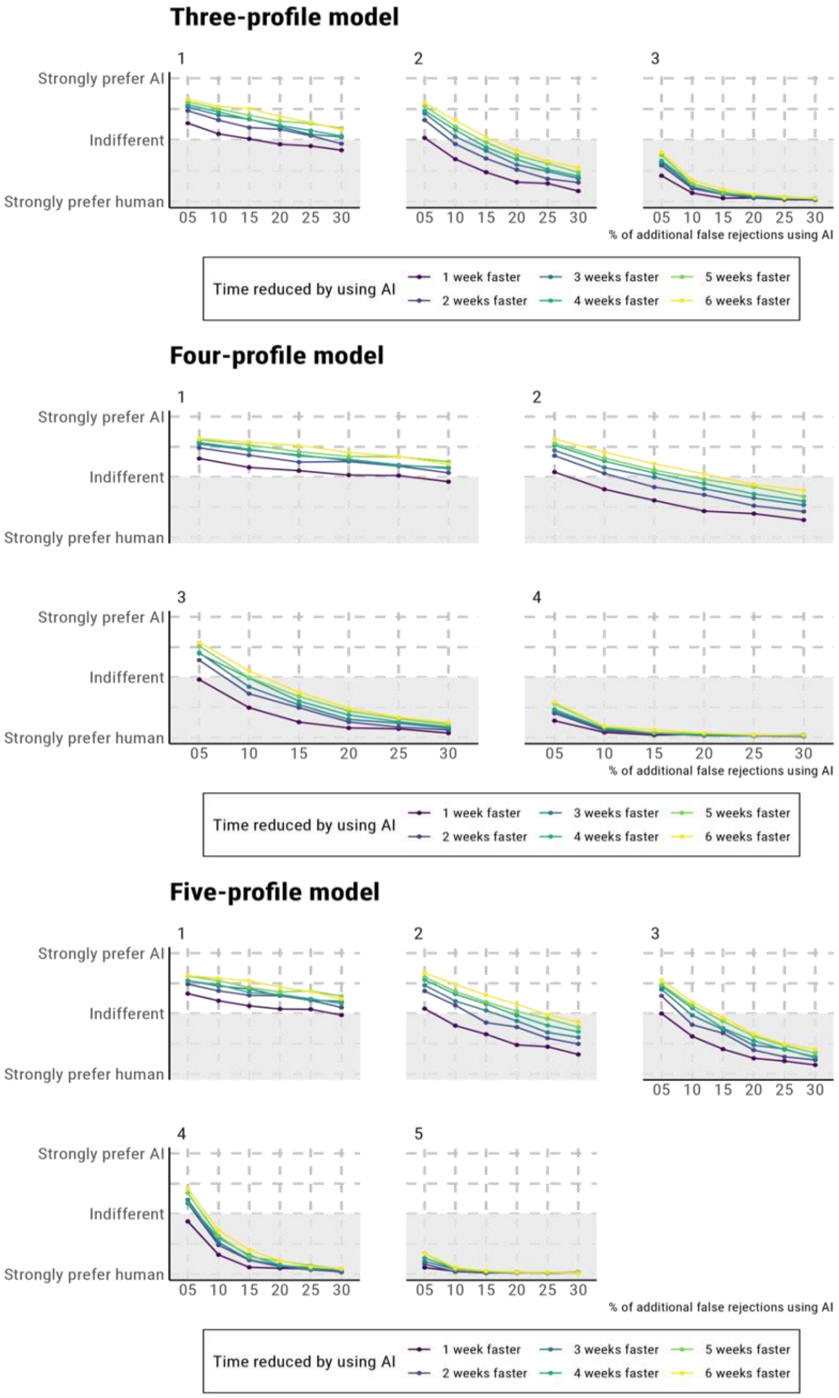

Supplementary Table 3.

Model parameters of the latent profile analysis in the UK balanced-sample study (*N* = 739).

Condition	Model with *N* of profile(s)	1 profile	2 profiles	3 profiles	4 profiles	5 profiles
All UK participants from own perspective (*N* = 739)	LogLik	-134948	-129678	-127250	-126364	-125466
	AIC	270056,8	259597,3	254824	253134,9	251420,6
	AWE	271191,6	261314,9	257124,2	256017,7	254886,1
	BIC	270425,2	260154,6	255570,1	254069,8	252544,3
	CAIC	270505,2	260275,6	255732,1	254272,8	252788,3
	CLC	269898,8	259357,3	254502	252730,8	250934,6
	KIC	270139,8	259721,3	254989	253340,9	251667,6
	SABIC	270171,2	259770,4	255055,7	253425,2	251769,6
	ICL	-270425	-260170	-255587	-254099	-252589
	Entropy	1	0,969854	0,974949	0,96861	0,962201
	prob_min	1	0,98992	0,988214	0,972457	0,970251
	prob_max	1	0,992559	0,993435	0,987896	0,998253
	n_min	1	0,466847	0,146143	0,077131	0,075778
	n_max	1	0,533153	0,465494	0,441137	0,339648
	BLRT_val	N/A	10541,43	4855,31	1771,145	1796,23
	BLRT_p	N/A	0,009901	0,009901	0,009901	0,009901
The UK participants from own perspective, without human redress (*N* = 369)	LogLik	-67831,2	-65134,5	-63777,7	-63205,9	-62897,5
	AIC	135822,4	130511	127879,4	126817,8	126283
	AWE	136846,1	132060,5	129954,6	129418,7	129409,5
	BIC	136135,2	130984,2	128513	127611,7	127237,3
	CAIC	136215,2	131105,2	128675	127814,7	127481,3
	CLC	135664,4	130271	127557,4	126413,8	125797
	KIC	135905,4	130635	128044,4	127023,8	126530
	SABIC	135881,4	130600,3	127999	126967,7	126463,1
	ICL	-136135	-130991	-128524	-127629	-127253
	Entropy	1	0,971923	0,971923	0,96685	0,97245
	prob_min	1	0,989401	0,966376	0,96725	0,96523
	prob_max	1	0,995024	0,996054	0,994699	0,985951
	n_min	1	0,490515	0,214092	0,116531	0,086721
	n_max	1	0,509485	0,487805	0,425474	0,382114
	BLRT_val	N/A	5393,353	2713,563	1143,595	616,8335
	BLRT_p	N/A	0,009901	0,009901	0,009901	0,009901
The UK participants from own perspective, with human redress	LogLik	-67046,7	-64451,7	-63286,4	-62812,2	-62524,8
	AIC	134253,4	129145,4	126896,7	126030,4	125537,6
	AWE	135277,5	130695,5	128972,7	128632,4	128665,5

($N = 370$)	BIC	134566,4	129618,9	127530,7	126824,9	126492,5
	CAIC	134646,4	129739,9	127692,7	127027,9	126736,5
	CLC	134095,4	128905,3	126574,7	125626,4	125051,6
	KIC	134336,4	129269,4	127061,7	126236,4	125784,6
	SABIC	134312,6	129235,1	127016,7	126180,8	125718,4
	ICL	-134566	-129627	-127540	-126840	-126514
	Entropy	1	0,968884	0,98031	0,966352	0,964623
	prob_min	1	0,985708	0,984636	0,973424	0,939855
	prob_max	1	0,994577	0,994943	0,997829	0,993243
	n_min	1	0,443243	0,124324	0,121622	0,059459
	n_max	1	0,556757	0,459459	0,389189	0,37027
	BLRT_val	N/A	5189,946	2330,706	948,2882	574,784
	BLRT_p	N/A	0,009901	0,009901	0,009901	0,009901

Supplementary Table 4.

Demographic descriptive information of 3 to 5 latent groups for the UK balanced-sample study (N = 739).

		Age	Sex (as females)	Income Mean (SD)	Welfare status (as claimants)
Three-profile model	Average	37.5 (11.2)	57.0%	4.9 (2.6)	47.1%
	Group 1 (39%)	37.0 (10.8)	53.7%	5.4 (2.6)	37.6%
	Group 2 (46%)	37.6 (11.8)	59.0%	4.7 (2.5)	52.0%
	Group 3 (15%)	38.2 (10.4)	59.3%	4.4 (2.6)	56.5%
Four-profile model	Average	37.5 (11.2)	51.4%	4.9 (2.6)	47.1%
	Group 1 (30%)	37.3 (10.5)	53.1%	5.3 (2.6)	38.4%
	Group 2 (44%)	36.8 (11.4)	58.3%	5.0 (2.5)	48.2%
	Group 3 (18%)	39.5 (12.0)	61.4%	4.5 (2.6)	53.0%
	Group 4 (8%)	36.9 (10.6)	54.4%	4.2 (2.4)	61.4%
Five-profile model	Average	37.5 (11.2)	51.4%	4.9 (2.6)	47.1%
	Group 1 (16%)	38.5 (10.9)	50.0%	5.5 (2.7)	33.6%
	Group 2 (28%)	36.4 (10.8)	55.8%	5.2 (2.6)	43.2%
	Group 3 (34%)	37.2 (11.5)	61.0%	4.8 (2.5)	50.6%
	Group 4 (15%)	39.2 (11.6)	59.1%	4.5 (2.7)	52.7%
	Group 5 (7%)	37.0 (10.7)	53.6%	4.1 (2.4)	62.5%

Supplementary Fig. 7.

Elbow plot for model selection in the UK balanced-sample study (N = 739).

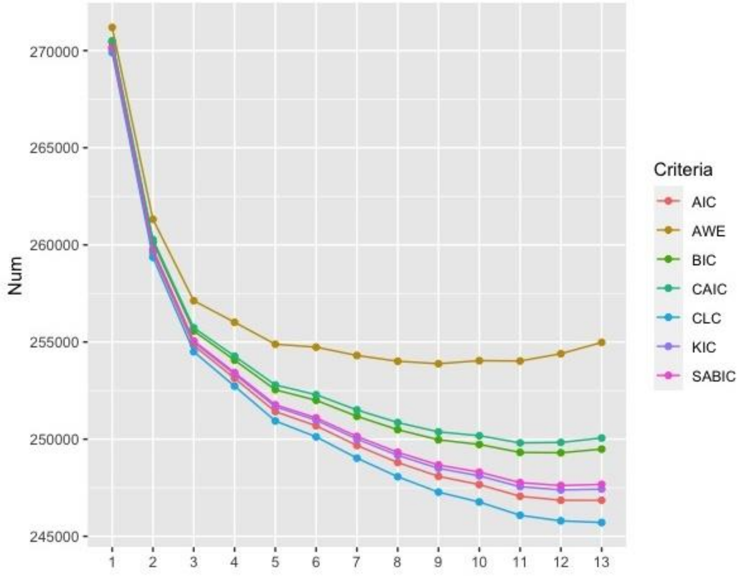

Supplementary Fig. 8.

Preference data clustered by latent profiles in the UK balanced-sample study ($N = 739$; the number of latent profiles ranged from 3 to 5).

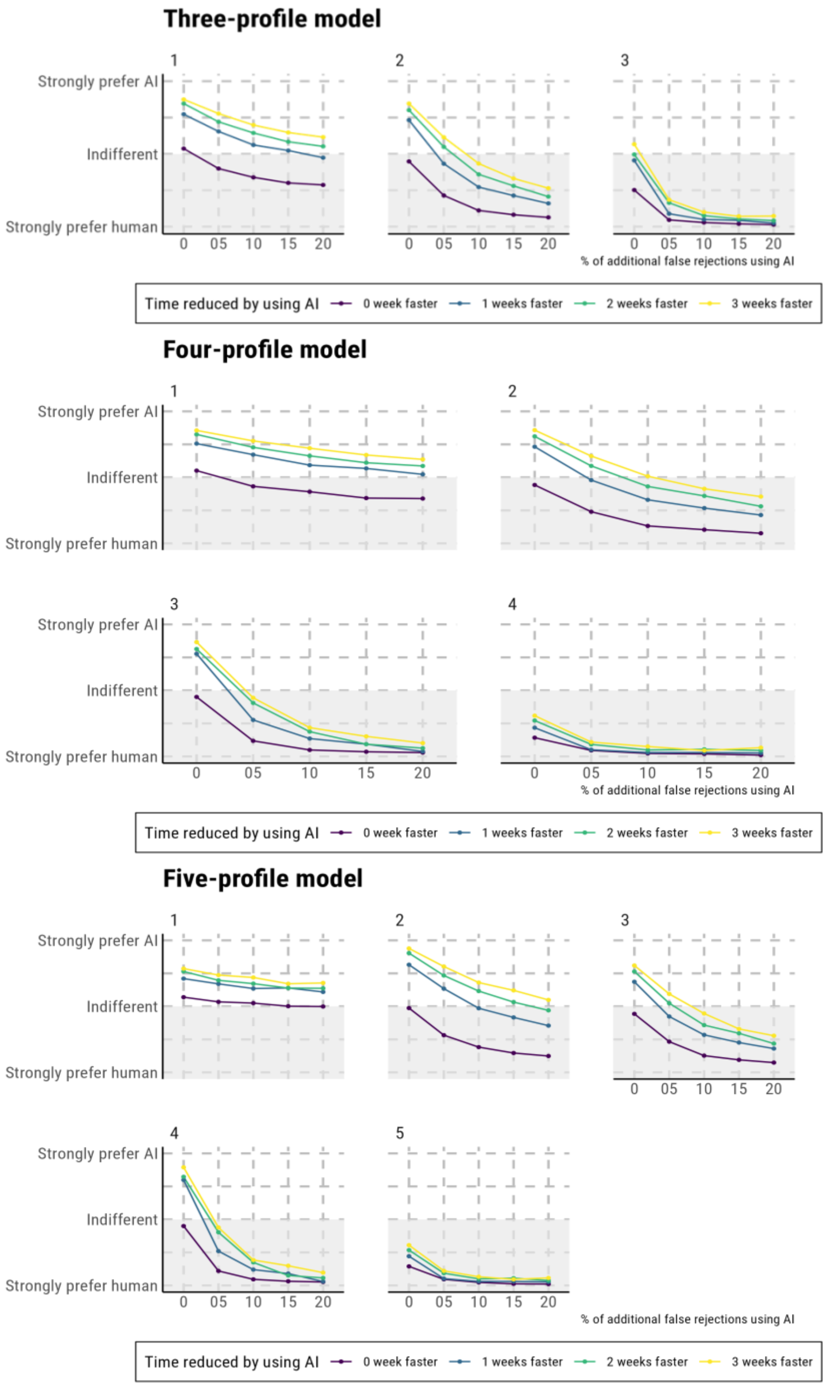

Supplement: Supplementary file 1 — Supplementary Information [file 41467_2025_62440_MOESM1_ESM.pdf]
