# Supplementary material for: Heterogeneous preferences and asymmetric insights for AI use among welfare claimants and non-claimants
Source: Nat Commun. 2025 Jul 29;16:6973. doi: 10.1038/s41467-025-62440-3 (PMC12307907; doi:10.1038/s41467-025-62440-3)
Supplement: Supplementary file 2 — Reporting Summary [file 41467_2025_62440_MOESM2_ESM.pdf]

nature portfolio

Corresponding author(s): Mengchen Dong

Last updated by author(s): May 30, 2025

Reporting Summary

Nature Portfolio wishes to improve the reproducibility of the work that we publish. This form provides structure for consistency and transparency in reporting. For further information on Nature Portfolio policies, see our Editorial Policies and the Editorial Policy Checklist.

Statistics

For all statistical analyses, confirm that the following items are present in the figure legend, table legend, main text, or Methods section.

n/a	Confirmed	
☐	☒	The exact sample size (*n*) for each experimental group/condition, given as a discrete number and unit of measurement
☐	☒	A statement on whether measurements were taken from distinct samples or whether the same sample was measured repeatedly
☐	☒	The statistical test(s) used AND whether they are one- or two-sided<br>*Only common tests should be described solely by name; describe more complex techniques in the Methods section.*
☒	☐	A description of all covariates tested
☐	☒	A description of any assumptions or corrections, such as tests of normality and adjustment for multiple comparisons
☐	☒	A full description of the statistical parameters including central tendency (e.g. means) or other basic estimates (e.g. regression coefficient) AND variation (e.g. standard deviation) or associated estimates of uncertainty (e.g. confidence intervals)
☐	☒	For null hypothesis testing, the test statistic (e.g. *F*, *t*, *r*) with confidence intervals, effect sizes, degrees of freedom and *P* value noted<br>*Give P values as exact values whenever suitable.*
☒	☐	For Bayesian analysis, information on the choice of priors and Markov chain Monte Carlo settings
☐	☒	For hierarchical and complex designs, identification of the appropriate level for tests and full reporting of outcomes
☐	☒	Estimates of effect sizes (e.g. Cohen's *d*, Pearson's *r*), indicating how they were calculated

Our web collection on statistics for biologists contains articles on many of the points above.

Software and code

Policy information about availability of computer code

Data collection	All studies were programmed and hosted on Qualtrics. All participants were recruited on Prolific.
Data analysis	All statistical analyses were conducted using R (version 4.3.1) within the RStudio environment (version 2024.09.1). The following R packages were used: tidyverse (version 2.0.0), lme4 (version 1.1.35.5), lmerTest (version 3.1.3), effectsize (version 1.0.0), margins (version 0.3.28). All code necessary to reproduce all analyses is openly accessible in the Open Science Framework repository, https://doi.org/10.17605/OSF.IO/Z637M.

For manuscripts utilizing custom algorithms or software that are central to the research but not yet described in published literature, software must be made available to editors and reviewers. We strongly encourage code deposition in a community repository (e.g. GitHub). See the Nature Portfolio guidelines for submitting code & software for further information.

Data

Policy information about availability of data

All manuscripts must include a data availability statement. This statement should provide the following information, where applicable:

- Accession codes, unique identifiers, or web links for publicly available datasets
- A description of any restrictions on data availability
- For clinical datasets or third party data, please ensure that the statement adheres to our policy

The datasets generated and analyzed during the current study are available in the Open Science Framework repository, https://tinyurl.com/welfareAI.

Research involving human participants, their data, or biological material

Policy information about studies with human participants or human data. See also policy information about sex, gender (identity/presentation), and sexual orientation and race, ethnicity and racism.

Reporting on sex and gender	Participants' sex was determined based on self-report. Sex was considered in the study design to ensure demographic representativeness or a sex-balanced sample. Data deaggregated by sex are provided in the Supplementary Notes B.1 to B.3, with consent obtained for reporting and sharing anonomyized individual-level data.
Reporting on race, ethnicity, or other socially relevant groupings	Ethnicity was measured in the study and was determined based on self-reports. Overall ethnicity distributions are reported in the Methods section of the manuscript. This information was not used in the main analyses.
Population characteristics	Age, sex, and ethnicity information were reported in the Methods section.
Recruitment	Participants were recruited using the online research recruitment platform Prolific. With the studies being titled "Artificial Intelligence in Welfare", it may have unintentionally attracted participants who have strong interests in AI or welfare.
Ethics oversight	All three studies complied with relevant ethical regulations for human subjects, were approved by the ethics committee at the Max Planck Institute for Human Development (NO. A2022-01, A2022-18, and A2024-16), and obtained informed consent from all participants.

Note that full information on the approval of the study protocol must also be provided in the manuscript.

Field-specific reporting

Please select the one below that is the best fit for your research. If you are not sure, read the appropriate sections before making your selection.

☐ Life sciences ☒ Behavioural & social sciences ☐ Ecological, evolutionary & environmental sciences

For a reference copy of the document with all sections, see nature.com/documents/nr-reporting-summary-flat.pdf

Behavioural & social sciences study design

All studies must disclose on these points even when the disclosure is negative.

Study description	Quantitative experimental
Research sample	One study used a US sample representative on country-level age, sex, and ethnicity distribution, and two other studies used US or UK samples with a balanced composition regarding sex and welfare claimant status. The samples were carefully chosen to address different aspects of the research questions.
Sampling strategy	Participants were randomly assigned into different welfare perspective conditions, and were repeatedly exposed to different welfare AI information. Sample sizes were determined using rule-of-thump recommendations, power simulations and systematic power analysis. Details are reported in the Methods section of the manuscript.
Data collection	Data were collected through a link released on Prolific. Participants were then directed to the Qualtrics interface and completed the studies online. Data collection was implemented by the first author who was aware of the experiment designs.
Timing	Data for the three studies were collected in February 2022, September 2022, and December 2024, respectively.
Data exclusions	Data exclusion only happened in the second UK study. As pre-registered, we filtered out 138 participants who provided different answers to one identical welfare status question ("Are you a recipient of Universal Credit?"; Answer: "Yes/No").
Non-participation	There were respectively 105, 181, and 117 drop-outs in the three studies. Data of participants who dropped out without completing the study were not included or used in the analyses.
Randomization	Participants, as either welfare claimants or non-claimants, were randomly assigned into different welfare perspective conditions.

Reporting for specific materials, systems and methods

We require information from authors about some types of materials, experimental systems and methods used in many studies. Here, indicate whether each material, system or method listed is relevant to your study. If you are not sure if a list item applies to your research, read the appropriate section before selecting a response.

Materials & experimental systems

n/a	Involved in the study
☒	☐ Antibodies
☒	☐ Eukaryotic cell lines
☒	☐ Palaeontology and archaeology
☒	☐ Animals and other organisms
☒	☐ Clinical data
☒	☐ Dual use research of concern
☒	☐ Plants

Methods

n/a	Involved in the study
☒	☐ ChIP-seq
☒	☐ Flow cytometry
☒	☐ MRI-based neuroimaging

Plants

Seed stocks	n/a
Novel plant genotypes	n/a
Authentication	n/a
